# Linking Social Prescribing and Preventive Occupational Therapy: A Way to Advance Health and Equity?

**DOI:** 10.1093/geroni/igaf122.775

**Published:** 2025-12-31

**Authors:** Marie-Hélène Lévesque, Mya Fischer, Mélanie Levasseur

**Affiliations:** Université de Sherbrooke, Sherbrooke, Quebec, Canada; Bishop’s University, Sherbrooke, Quebec, Canada; Université de Sherbrooke, Sherbrooke, Quebec, Canada

## Abstract

Presented as a response to unmet social needs and a way to foster a more equitable, sustainable, and healthy society, social prescribing (SP) faces challenges and criticisms, and little is known about its compatibility with preventive occupational therapy, particularly the Lifestyle Redesign program (LR), a landmark intervention with shared principles. To explore this compatibility this narrative umbrella review draws on seven systematic reviews supplemented by expert contributions from the National Academy of Social Prescribing. Results indicate conflicting evidence of SP on individuals, communities and healthcare systems. Despite widespread implemented in the UK and gaining traction globally, including in Canada and U.S., few peer-reviewed studies have examined its effects on older adults, and those available are of limited quality. Most studies reported positive changes in health self-management, loneliness, social isolation, and well-being, with high acceptability among participants and general practitioners. The COVID-19 pandemic highlighted both the importance of social prescribing in addressing these issues and the challenges of its implementation during crises. SP remains underutilized by occupational therapists, even though their expertise aligns with its objectives. Strengthening SP-LR linkages could advance interdisciplinary collaboration through three approaches: (1) LR as an activity within SP, (2) a navigator dedicated to LR, and (3) the occupational therapist as navigator. While not a unique solution, integrating SP within occupational therapy practice could improve access to preventive interventions, notably for older adults at risk of loneliness, social isolation, and chronic disease, and empower clinicians to address social determinants of health and drive innovation in gerontology.

